# Coagulopathy and acute pancreatitis: pathophysiology and clinical treatment

**DOI:** 10.3389/fimmu.2024.1477160

**Published:** 2024-10-31

**Authors:** Lan Li, Qingyuan Tan, Xueying Wu, Xiaowen Mou, Ziqi Lin, Tingting Liu, Wei Huang, Lihui Deng, Tao Jin, Qing Xia

**Affiliations:** ^1^ West China Center of Excellence for Pancreatitis, Institute of Integrated Traditional Chinese and Western Medicine, West China Hospital, Sichuan University, Chengdu, China; ^2^ Department of Integrated Traditional Chinese and Western Medicine, West China Tianfu Hospital, Sichuan University, Chengdu, China; ^3^ West China Biobank, West China Hospital, Sichuan University, Chengdu, China

**Keywords:** coagulopathy, acute pancreatitis, immunothrombosis, pathophysiology, clinical treatment

## Abstract

Coagulopathy is a critical pathophysiological mechanism of acute pancreatitis (AP), arising from the complex interplay between innate immune, endothelial cells and platelets. Although initially beneficial for the host, uncontrolled and systemic activation of coagulation cascade in AP can lead to thrombotic and hemorrhagic complications, ranging from subclinical abnormalities in coagulation tests to severe clinical manifestations, such as disseminated intravascular coagulation. Initiation of coagulation activation and consequent thrombin generation is caused by expression of tissue factor on activated monocytes and is ineffectually offset by tissue factor pathway inhibitor. At the same time, endothelial-associated anticoagulant pathways, in particular the protein C system, is impaired by pro-inflammatory cytokines. Also, fibrin removal is severely obstructed by inactivation of the endogenous fibrinolytic system, mainly as a result of upregulation of its principal inhibitor, plasminogen activator inhibitor type 1. Finally, increased fibrin generation and impaired break down lead to deposition of (micro) vascular clots, which may contribute to tissue ischemia and ensuing organ dysfunction. Despite the high burden of coagulopathy that have a negative impact on AP patients’ prognosis, there is no effective treatment yet. Although a variety of anticoagulants drugs have been evaluated in clinical trials, their beneficial effects are inconsistent, and they are also characterized by hemorrhagic complications. Future studies are called to unravel the pathophysiologic mechanisms involved in coagulopathy in AP, and to test novel therapeutics block coagulopathy in AP.

## Introduction

The activation of coagulation by inflammation cascade are essential reactions for host defense during inflammatory diseases (1). Pathogen-associated molecular patterns (PAMPs) and damage associated molecular patterns (DAMPs) are recognized by pattern-recognition receptors on the cells of the innate immune system, which triggers the release of pro-inflammatory mediators (2). Pro-inflammatory mediators then activate the coagulation cascade, downregulate crucial endogenous anticoagulant mechanisms, and dysregulate fibrinolytic mechanisms. In turn coagulation disorders also markedly influences inflammatory response (2). The primitive response represents an effective strategy to slow inflammatory storm spread and maintain hemostasis, while this may come at the cost of immune-driven pathological thrombus formation, which is now commonly termed ‘immunothrombosis’ (3).

In acute pancreatitis (AP), one of the early events is the pancreas autodigestion due to premature trypsinogen activation (4). Injured acinar cells release cytokines, chemokines, and adhesion molecules into the circulatory system, which recruit the infiltration of immune cells to the site of injuries and initiate coagulation (5). Histologic evaluation of AP indeed shows inflammatory cell infiltration, elevated circulating tissue factor (TF), platelet aggregation, intravascular microthrombi, fibrin deposits (6–8) and microcirculation hypoperfusion of extrapancreatic organs (9, 10). From a clinical perspective, coagulation disorders are common in patients with severe AP, with severity ranging from clinically less apparent microvascular clot formation to devastating thrombotic and hemorrhagic complications (11–14).

Despite recognizing the potential deleterious of coagulopathy on the outcome in severe AP patients, effective treatments specifically aiming to block the devastating complications while maintaining its beneficial effects for the host, do not yet exist. Although a variety of anticoagulants drugs have been evaluated in clinical trials, their beneficial effects are inconsistent, and they are also characterized by a high rate of hemorrhage complication. Severe AP patients with coagulopathy, particular with disseminated intravascular coagulation (DIC) are at a higher risk for persistent organ failure and pancreatitis-associated death (15), hemorrhage complication may bring these patients into life threatening situation. International guidelines therefore discourage anticoagulant therapies in severe AP cases (16). Nowadays, there is still ongoing research assessing the effect of new molecules on thrombosis in severe AP, with agents targeting intracellular inflammatory pathways, P-selectin and neutrophil extracellular traps (NETs) formation demonstrating promising results. A better understanding of the underlying mechanisms and cellular interactions in AP-related immunothrombosis and coagulopathy is crucial to identifying new therapeutic targets. Our study summarizes the current literature regarding the role of innate immune cells, endothelial cells and platelet in coagulopathy in AP and summary clinical evidence on drugs targeting the critical pathological process.

## Pathology of clinically relevant coagulopathy in acute pancreatitis

### Role of monocytes and tissue factor in the coagulopathy of AP

**Figure 1:**
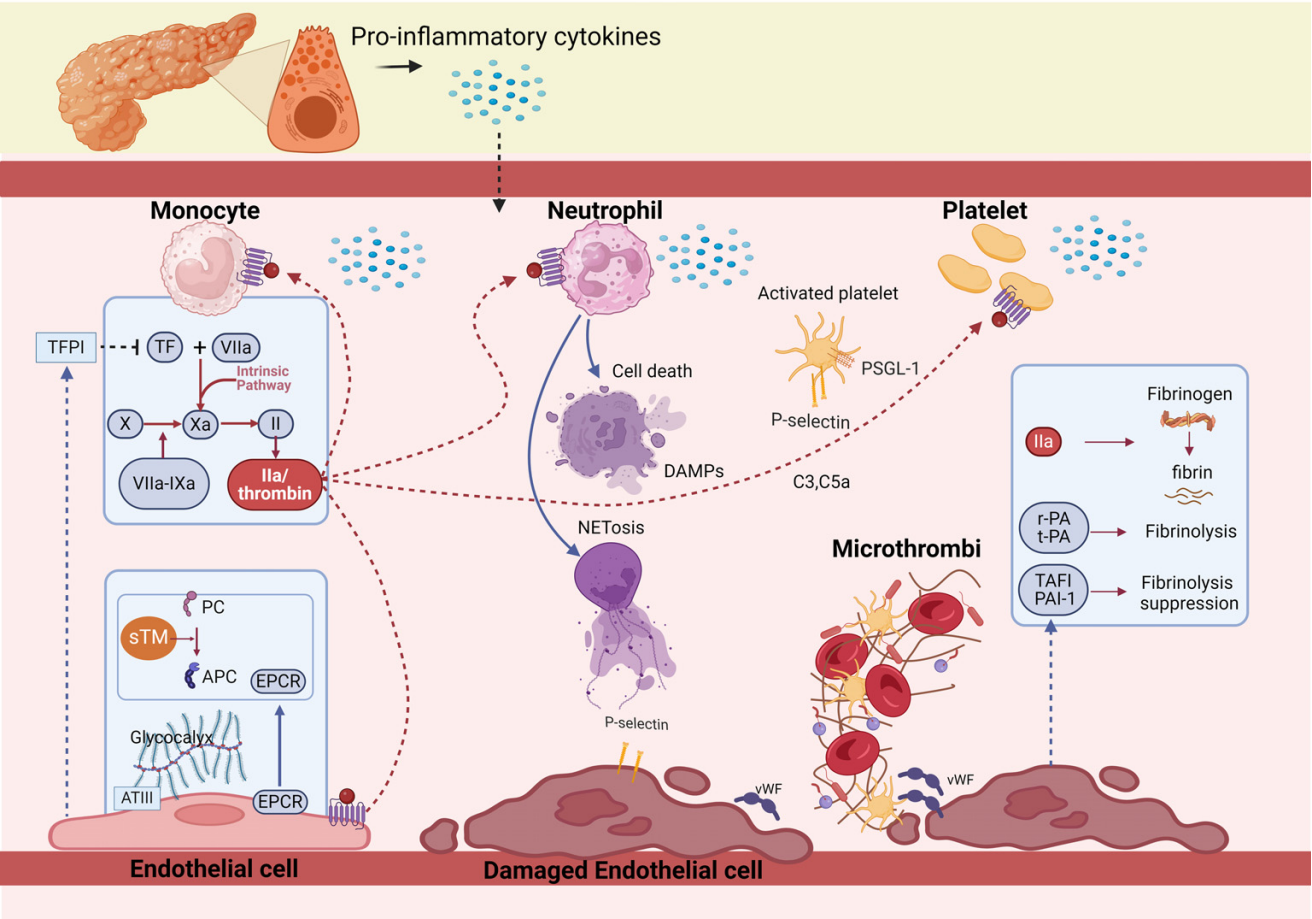
Pathophysiology of coagulopathy in acute pancreatitis. The activation of coagulation by inflammation cascade are essential reactions for host defense during acute pancreatitis. Initiation of coagulation activation is caused by expression of tissue factor on activated monocytes and is ineffectually offset by tissue factor pathway inhibitor. TF expression and release triggers the extrinsic coagulation pathway by binding factor VII/factor VIIa to form TF-FVIIa complex, converting factor X to factor Xa. FXa, as the prothrombinase then, thrombin is formed. At the same time, endothelial-associated anticoagulant pathways, in particular the protein C system, which includes PC, Thrombomodulin and endothelial cell protein C receptor, is impaired by pro-inflammatory cytokines. Also, fibrin removal is severely obstructed by inactivation of the endogenous fibrinolytic system, mainly as a result of upregulation of its principal inhibitors, plasminogen activator inhibitor type 1 and thrombin activated fibrinolytic inhibitor. Finally, increased fibrin generation and impaired break down lead to deposition of (micro) vascular clots.

Monocytes and macrophages have been found to play a vital role in inflammatory diseases-induced immunothrombosis (**Figure 1**). Upon stimulation by PAMPs, DAMPs or proinflammatory mediators (17, 18), monocytes are the main source of circulating TF (19, 20). TF is deemed critical for survival, as deletion in mice leads to universal embryonic death (21), and defects in TF gene expression are associated with differing clinical outcomes in patients with sepsis (22). The binding of lipopolysaccharide to transmembrane receptors in monocytes induces TF mRNA expression via NF-κB activation (23). The interaction of pathogen components either with TLRs or directly with intracellular pathways in monocytes result in inflammasome activation and subsequent TF release via pyroptosis (18, 23, 24). Moreover, pore formation on the cell membrane also induce calcium influx, which triggers phosphatidylserine exposure on the membrane, followed by TF activation (25). Sphingomyelin, another membrane lipid, is also involved in the activation of TF to its procoagulant form (26). Additionally, monocyte activation by PAMPs and DAMPs is followed by increased P-selectin glycoprotein ligand 1 (PSGL-1) expression and the release of TF- and PSGL-1-bearing microparticle (MPs). These MPs can fuse *in vitro* with platelets, leading to increased TF activity (19, 27). Pancreatic disruption leads to direct exposure of TF to the blood (28).

TF expression and release triggers the extrinsic coagulation pathway by binding factor VII/factor VIIa (FVII/FVIIa) to form TF-FVIIa complex, converting factor X (FX) to factor Xa (FXa). Then, FXa is incorporated into FXa-factor Va-Ca2+-phospholipids (FXa-FVa-Ca2+-PLs) complex known as the prothrombinase (29). Thrombin is formed, leading to fibrin clots (**Figure 1**). The process seems to be the most potent pathway leading to coagulation cascade activation and DIC (30–34). Studies of experimental or human AP have demonstrated a central role of the TF/FVIIa system in the initiation of thrombin generation (8, 35, 36). In the early stage of severe AP, TF is highly upregulated (8, 37–39), and it is a favorable predictive marker of severe AP (40, 41). Abrogation of the TF/FVIIa pathway by specific interventions aimed at TF or factor VIIa activity resulted in a complete abrogation of thrombin generation in experimental settings (42). As the initiator of the coagulation cascades, TF might play a large part in the development of severe AP, there needs to be a more basic experimental to explore their relationship.

### Role of endothelial cells in the coagulopathy of AP

The endothelium lines the lumen of the entire circulatory system, separating blood and subendothelial, and maintaining vascular health by exerting anticoagulant action via tissue factor pathway inhibitor (TFPI), Protein C (PC) system, Antithrombin (ATIII) and fibrinolysis (43, 44).

### TFPI

TFPI is the inhibitor of TF-mediated coagulation, primarily synthesized by endothelial cells (ECs, **Figure 1**), which binds to ECs via proteoglycans/glycosaminoglycans, inactivates TF-FVIIa-FXa complex and prothrombinase in the early phase of the coagulation process (45, 46). The deficiency of TFPI increases susceptibility to the development of DIC and thrombosis (37). However, in AP patients, the plasma TFPI levels were significantly increased, which might be compensatory to the rise of TF, and can be released from fibrin deposits after thrombosis. Elevation of TFPI delayed TF-initiated thrombin generation, the imbalance of TF/TFPI were markedly related to pancreatic necrosis and organ failure (OF) (38, 47).

### PC system

The PC system, as main natural anticoagulants, harbors PC and Thrombomodulin (TM), which along with endothelial cell protein C receptor (EPCR) catalyzes the thrombin-mediated PC activation (**Figure 1**). Activated PC (APC) exerts potent anticoagulation by inactivating FVa and FVIIIa (48). TM is expressed on the endothelial surface, which switches the thrombotic activity of thrombin to antithrombotic through activating PC (49, 50). It is known that soluble TM (sTM), fragments of the extracellular region of membrane-bound TM cleaved by leukocyte-derived proteases or metal loproteases, are released into the circulation in inflammatory diseases (50). Multiple studies have reported the usefulness of measuring sTM to evaluate the severity of DIC. EPCR, a transmembrane glycoprotein present on the surface of ECs, increases the efficiency of APC generation by presenting PC zymogen to thrombin/TM complex (51). However, the PC system is damaged in AP patients characterized by low levels of PC and APC (52, 53), and significantly increased levels of plasma sTM and EPCR (54).

### ATIII

ATIII, as a serine protease inhibitor, which inactivates TF-FVIIa-FXa Complex, FIXa, FXIa, thrombin, and is the most abundant and most important physiological anticoagulant (55). ATIII makes complexes not only with thrombin but also bind to heparan sulfate of the glycocalyx at the ECs surface (56) (**Figure 1**). ATIII deficiency can result in severe venous thromboembolism, plasma levels of ATIII activity is positively correlated with the severity of DIC (57). In AP patients, the level of ATIII decreases as severity increases, which is rather pronounced in cases of biliary AP (52). This phenomenon could be ascribed to a combination of impaired synthesis because of the negative acute phase response, degradation by elastase, and consumption because of thrombin generation (37, 58).

### Fibrinolysis

Tissue-type plasminogen activator (t-PA) and urokinase-type PA (u-PA) released by ECs are the main activators in the fibrinolysis, which transform plasminogen into plasmin, and then catalyze clot dissolution and fibrinolysis. PA inhibitor, type 1 (PAI-1) and thrombin activated fibrinolytic inhibitor (TAFI) are the regulators of the fibrinolysis, of which PAI-1 is the principal inhibitor (59) (**Figure 1**). It has been shown that the production of PAI-1 is affected by proinflammatory, anti-inflammatory cytokines and the elevated levels sustain longer (60). In healthy volunteers, endotoxin induces a rapid activation in the coagulation system with a concurrent rise in tPA. This temporal activation in fibrinolysis is subsequently counteracted by a greater and sustained rise in PAI-1 (61), The marked increase in PAI-1 level causes fibrinolysis shutdown, subsequently failing to counteract the systemic deposition of fibrin clots during system inflammatory reaction syndrome, leading to thrombosis and DIC (59). Patients with OF have significantly higher plasma levels of PAI-1, and non-survivors demonstrate more potent suppression of fibrinolysis than survivors (36). In severe AP, the level of TAFI also rises at the onset of the disease (58), inhibits fibrinolysis by separating carboxyterminal lysine residues and preventing binding to plasminogen (62).

### Role of platelets and P-selectin in the coagulopathy of AP

Cytokines (63) and thrombin (64–67) activate platelets by DAMP receptors (68) (**Figure 1**), myeloid differentiation factor 88 (MyD88) and cGMP-dependent protein kinase intracellular pathways (67), as well as protease associated receptors (64, 65). Upon platelet activation, dense and α-granules fuse with the cell membrane (65), dense granules are rich in adenosine diphosphate, which further stimulates and amplifies platelet activation via receptors P2Y1 and P2Y12, whilst α-granules contain P-selectin that mediates activation of leukocytes via binding to PSGL-1, chemokines, and pro-coagulant factors. Glycoproteins IIb/IIIa (GPIIb/IIIa) and Iba (GPIba), expressed on the surface of activated platelets (69), bridged by fibrinogen or von Willebrand Factor (vWF), which constitute another receptor category that promotes platelet degranulation and aggregation (69, 70). VWF, from α-granules, facilitate platelet adhesion to the endothelium (71) (**Figure 1**). FcgRIIa triggers an intracellular pathway for platelet activation by phosphorylating the tyrosine kinases Src, Syk, and phospholipase c gamma 2 (PLCg2) (70). Mechanical interactions are potentiated by change from discoid to stellate shape (72). Activated platelets have also been found to release polyphosphate (PolyP), an inorganic polymer that exerts procoagulant activity. *In vitro*, PolyP initiates the contact pathway by FXII activation (73). Further, activated platelets aggregate with leucocytes to form platelet-leucocyte aggregates (PLA) (74), PLA in turn cause release of platelets-activating neutrophil extracellular traps (NETs), which form a vicious cycle. Platelets are essential cellular components of the coagulation system in AP animal models (8). In AP, thrombocytopenia is associated with increased disease severity and an ominous prognosis (75).

### P-selectin

P-selectin stored in granular structures of ECs and platelets can be quickly mobilized towards the cell surface upon stimulation (76). P-selectin and its ligand, PSGL-1 linking is the first step for platelet adhesion (56) (**Figure 1**). PSGL-1 expressed on platelets, monocytes, and neutrophils mediate leukocyte and platelet rolling on the vascular wall as well as platelet-neutrophil and platelet–platelet aggregations to link inflammatory infiltration and thrombus formation (77). The expression of P-selectin on the platelet membrane not only mediates the adherence of platelets to leukocytes and endothelial cells but also enhances the expression of TF on monocytes (78). Notably, monocyte-derived, TF containing MPs fail to incorporate in thrombi when infused into P-selectin null mice, indicating that the accumulation of leukocyte-derived TF in growing thrombi is mediated by PSGL-1 on the MPs (79).

The levels of P-selectin are related to the development and course of AP, it’s value on admission may play a pivotal role as indicators of overall prognosis (80). The elevated level of P-selectin markedly strengthens the leukocyte–endothelium interaction and the thrombosis (81). Suppressing P-selectin inhibits leukocyte and platelet rolling in postcapillary venules of the inflamed pancreas (82), protecting against thrombosis (83) and improving pancreatic microcirculation and histopathology of acinar necrosis without causing any bleeding complications (84). Escopy et al. reviewed both preclinical and clinical trials that have evaluated therapeutic potential of biologic and small-molecule inhibitors as well as antibodies of P-selectin in a variety of diseases linked to immunothrombosis and coagulopathy (85). Wherein, crizanlizumab, a monoclonal antibody of P-selectin, has been evaluated for the treatment of vaso-occlusive crises with sickle cell disease (Food and Drug Administration approval in 2019) and COVID-19 vasculopathy (NCT04435184 and NCT04505774). Inclaclumab, a newly developed monoclonal antibody of P-selectin, was noted to reduce myocardial damage of non-ST-elevation in patients with myocardial infarction in a phase 2 clinical trial (NCT01327183). Currently, a multicenter phase 3 trial is in progress to determine whether inclaclumab could reduce the frequency of vaso-occlusive crises (Thrive-131; NCT04935879) and to evaluate its long-term safety (Thrive-133 open-label extension; NCT05348915). Except for these anti-P-selectin antibodies, PSI-697, PSI-421, as small-molecule inhibitors of P-selectin, have also been widely studied (85, 86). P-selectin and P-selectin glycoprotein ligand-1 play a fundamental role in aggravating pancreatic inflammation and their antibodies alleviate inflammatory responses in experimental severe AP (82). Therefore, it is believed that further research on the therapeutic potential of these inhibitors of P-selectin and related pathways in severe AP maybe promising.

### Role of neutrophil and NETs in the coagulopathy of AP

DAMPs also activate neutrophils, which are typically the first responders to AP. Activated neutrophils exert their antimicrobial activity mainly through three processes: phagocytosis, degranulation, and the release of NETs (87). Neutrophil activation and release of NETs are considered as the initial and indispensable event in thrombus formation (88) (**Figure 1**). NETs as a meshwork of DNA fibers, comprise histones, antimicrobial proteins, and high-mobility group box 1 (89), which promote endothelial dysfunction (90), increase platelet activation, adhesion, aggregation (89–91), in turn contribute to thrombin-mediated fibrin generation (92, 93). NETs also propagate thrombosis by capturing TF and TF-positive extracellular vesicles from the circulation, further driving coagulation (94). Wherein, thrombin formation consists of NETs-induced platelet-dependent mechanisms and platelet-poor plasma via activation of the intrinsic coagulation pathway (95). Interaction of NETs with membrane-derived MPs released by activated neutrophils further enhanced NET-mediated intrinsic coagulation pathway activation (96). However, the role of NET components, or intact NETs on thrombosis is debatable, and merit further investigation. Except for prothrombotic role, NETs were also shown to interfere with the endogenous anticoagulant mechanisms. More specifically, extracellular nucleosomes within NETs facilitated TFPI degradation by neutrophil elastase on the surface of activated neutrophils (97), neutrophil elastase bound to DNA complexes was also shown to cleave plasminogen into fragments, cell-free DNA was capable of binding to plasmin and fibrin at the same time, resulting in decreased plasmin production and impaired fibrinolysis (98). H3 and H4, could also interact with TM and PC, leading to the inhibition of APC generation (99).

In AP patients, the plasma levels of NET components increase significantly compared to the controls (100). Platelets regulate the formation of NETs and NET-MPs aggregations (100–102) and in turn, NETs recruit platelets and neutrophils, reinforcing each other and injuring the endothelium within pancreatic microvasculature (100, 103). Exosomes adhere to NETs *in vitro* where they have a dose-dependent pro-coagulant effect. NETs also activate the intrinsic coagulation pathway via autoactivation of Factor XII (102). NETs are complex structures composed of DNA and cytotoxic granule proteins, including myeloperoxidase and neutrophil elastase. NETs targeting, either preventing their formation or degrading the NETs that have already formed, has been proved to prevent tissue damage and reduce risk of thrombus formation in the context of infections (104). Preventing NETs formation by inhibition protein-arginine deaminase 4, neutrophil elastase, or gasdermin D, has been proved to be effective in several preclinical inflammatory disease models. While whether inhibition of NETs formation has a detrimental effect on host defense mechanisms has not been determined (105). Another strategy could be to interfere with NETs that have already formed: a recombinant human deoxyribonuclease I (rhDNase) is already used for the treatment of cystic fibrosis with safety confirmed, making it a very viable option for other diseases. A phase Ib study for patients with systemic lupus erythematosus (SLE) showed that DNase was well tolerated without severe adverse effects. Significant recent developments in the field of rhDNase targeting the NET have led to testing of new NET-targeted drugs in clinical trials of patients with COVID-19 (NCT04409925, NCT04541979 and NCT05139901) (105). Heparins, as a class of anticoagulant drugs, has been proposed to destabilize NETs by dissociating histones from the chromatin backbone of the extracellular traps as well as to prevent phorbol myristate acetate-induced NET formation (106). Colchicine, destabilization of actin cytoskeleton in NETs has been tested in Gout. N-acetyl cysteine, a ROS scavenger, improved the condition of patients with SLE and acute liver failure. Anti-TNF monoclonal antibodies have been used for the treatment of rheumatoid arthritis (RA) and inflammatory bowel disease, and anti-IL-17 antibodies have also shown some efficacy for the treatment of RA (107). The involvement of NETs has been well established in the pathobiology in experimental models and patients of severe AP (100, 108, 109). All the aforementioned therapeutic agents targeting NETs are expected to have significant potential to mitigate severe AP in transitional research (105).

### Role of complement system in the coagulopathy of AP

The complement system shares a common origin with the coagulation system and influences each other. It is activated through proteolytic cascades (110), leading to the formation of membrane attack complexes, ultimately polymerizing and inducing lysis of the cellular target (111). Recent studies have shown specific crosstalk between complement and coagulation in AP patients (112). First, in addition to activation by serine proteases, granzyme B and trypsin also cleave the central complement components, generating C3a and C5a (113). Second, C3a and C5a release TF from monocytes and ECs and promote platelet activation, leading to thrombogenesis (110, 111). Moreover, C3 is essential for the recruitment of neutrophils into the pancreas and NET formation (114). After C3a and C5a complement activation, the direct products also stimulate the platelets and promote coagulation by stimulating phosphatidylserine exposure (111) (**Figure 1**), enhancing the activation of platelets, granulocytes, and ECs, increasing the microcirculation thrombosis and pancreatic injury.

## Diagnosis, monitoring and potential biomarkers of the coagulopathy in acute pancreatitis

Early diagnosis and monitoring of coagulopathy is sometimes not straightforward and complicated in daily clinical practice. Among the items of the International Society on Thrombosis and Hemostasis (ISTH) score, fibrinogen concentrations and platelet counts might be increased in the early phase of AP because of inflammation (3), thrombocytopenia may also be due to other conditions, such as immune thrombocytopenia, heparin-induced thrombocytopenia, thrombotic microangiopathies, or medication-induced bone marrow depression (115), and poor sensitivity of the ISTH criteria for the diagnosis of DIC has been reported (116). Vitamin K deficit and liver insufficiency may also be present at the same time with AP associated coagulopathy, this differentiation is not always easy (117). Plasma D-dimer alone could predict coagulopathy and severity in patients with AP (118), while it might also be increased because of inflammation (119). It would be interesting to see whether new diagnostic criteria for DIC from the Japanese Society on Thrombosis and Hemostasis, which takes into account the underlying diseases (116). Thrombelastography is a viscoelastic assay that measures clotting of whole blood over time measured using a spinning wire probe, and is increasingly employed in severe AP patients with a hypercoagulable state (120). In total, early diagnosis and monitoring of coagulopathy in severe AP is still a challenge (15, 121), sequential assessment of fibrinogen (122), point-of-care tests (123) and biomarkers base on immunothrombosis might be more helpful and yield diagnostic insight (124). Further elucidation of the mechanisms of coagulopathy as well as the proper diagnostic criteria, and potential biomarkers would contribute to the improved management of prognosis of this intractable disease.

## Clinical evidence on drugs targeting the coagulopathy of AP

The efficacy of Food and Drug Administration (FDA)-approved drugs commonly used in clinical practice, such as heparin, has been fully tested AP patients by several small-scale clinical investigations (**Table 1**). In addition to FDA approved drugs, a variety of currently non-FDA-approved agents, including APC and rTM, have also been evaluated in AP (**Table 1**). According to the international guidelines, anticoagulant medications are not recommended for AP or severe AP, or AP patients coexisting DIC and splanchnic vein thrombosis (SVT) (146). Although the PC pathway defects are associated with the progress of multiple OF (53), the coagulation disorder in severe AP with APC treatment is not improved in patients (125), and the recovery from coagulopathy is slower than the placebo group (126). Studies assessing the efficacy of TM have shown more promising results, as rTM administration resulted in decreased mortality and was not associated with increased bleeding events in AP-induced coagulopathy (127–129). A recent study also showed that rTM effectively prevented the development of walled-off necrosis (127, 129). Similarly, low molecular weight heparin (LMWH) is not recommended in the initial managements of moderately severe AP and severe AP patients, although some studies have found a beneficial effect on OF, local complication, mortality, length of stay (LOS) without increase the risk of bleeding complications^112-116^. As for anticoagulation therapy in the AP patients complicated with SVT in the later stage, heparin or LMWH followed by warfarin or novel oral anticoagulant are not approved for clinical use in AP due to both inconclusive results for their efficacy (139, 140, 142–145, 147, 148) and an increased risk for bleeding side effects (139, 142). Carrying out larger, multicenter clinical trials designed to evaluate the potential treatment benefit of LMWH and rTM replacement in AP is encouraged.

**Table 1:** Selected clinically approved and pre-clinical therapeutics that may block coagulopathy in AP.

Inhibitor	Targets	Author	Year	Setting	Study Design	Patient Population	Intervention (patient number)	Comparator	Main findings
APC	FVa, FVIIIa, FII	Pettilä et al. (125)	2010	Tertiary (SC), Finland	Prospective double blind randomized pilot study	SAP	24 μg/kg/h for 96 hours (n=16)	Placebo (n=16)	No significant difference in MODS and OF-free days
		Kyhälä et al. (126)	2015	Tertiary (SC), Finland	Controlled study	SAP	24 μg/kg/h for 96 hours (n=10)	Placebo (n=10)	↓ Recovery from coagulopathy
rTM	PC	Eguchi et al. (127)	2015	Tertiary (SC), Japan	Retrospective survey	SAP	380 U/kg/d or 130 U/kg/d for SAP patients with DIC on hemodialysis, until DIC scores improved to a JAAM score of ≤3 (n=24)	Without early administration of rTM (n=30)	↓ WON
		Yano et al. (128)	2019	Tertiary (SC), Japan	Retrospective survey	SAP with DIC	Same as Eguchi et al. research (127) (n=13)	Same as Eguchi et al. research (127) (n=25)	↓ Platelet count, mortality on the 60th day
		Eguchi et al. (129)	2021	Tertiary (MC), Japan	Retrospective survey	AP with ANC and APFC	380 U/kg/d for SAP patients with DIC (n=18)	Same as Eguchi et al. research (127) (n=80)	↓ Risk of ANC developed WON.
LMWH	TF-FVIIa-FXa complex, FIIa, FIXa, FXIa	Lu et al. (130)	2009	Tertiary (MC), China	RCT	SAP	100 µg/kg/d for 7 days (n=135)	Conventional treatment (n=130)	↑ Clinical and laboratory data improvement; ↓ Mortality, LOS, CTSI
		Du et al. (131)	2014	Tertiary (SC), China	RCT	SAP	5,000 U b.i.d. (n=34)	Conventional treatment (n=33)	↓ MOF, surgery, mortality, LOS
		Tozlu et al. (132)	2019	Tertiary (SC), Turkey	Randomized, controlled, open-label study	MSAP with symptoms ≤ 24h	1 mg/kg b.i.d. for 7 days (n=50)	Conventional treatment (n=50)	↓ PN, local and systemic complications
		Li et al. (133)	2019	Tertiary (SC), China	Retrospective Study	MSAP and SAP	38000–76000 IU/d in patients with hypercoagulability (n=541)	Without anticoagulation (n=284)	No significant difference in sinistral portal hypertension
		Kröner et al. (134)	2020	NIS data, US	Case-control, retrospective study	AP patients with systemic anticoagulation	AP who were on anticoagulation, no distinction on type (n=7827)	Without anticoagulation (n=182, 647)	↓ OF, AKI, ICU, mortality ↑ LOS, costs
		Patil et al. (135)	2022	Tertiary (SC), India	Randomized, single blind, phase 3 control trial	MSAP	1mg/kg Enoxaperin b.i.d. for 7 days (n=70)	Conventional treatment (n=70)	↓ Chance of disease progression in CTSI and PN.
LMWH With warfarin or AC	FIX, FX, FVII, FII	Gonzelez et al. (136)	2011	Tertiary (SC), UK	Retrospective analysis of prospective data	AP with SVT	LMWH (1 mg/kg) subsequently warfarin, upon discharge (n=4)	Without anticoagulation (n=20)	No significant difference in recanalization rate
		Harris et al. (137)	2013	Tertiary (SC), US	Database	AP with SVT	Enoxaparin 1 mg/kg b.i.d. or ivgtt unfractionated heparin (initial bolus of 80 U/kg) followed by an initial infusion rate of 18/kg/h), subsequently, warfarin (n=17)	Without anticoagulation (n=28)	No significant difference in recanalization rate and mortality
		Easler et al. (138)	2014	Tertiary (MC), US	Retrospective study	AP with SVT	Anticoagulation (n=6)	Without anticoagulation (n=16)	Infrequent administration of anticoagulants; No complications directly related to SVT.
		Garret et al. (139)	2018	Tertiary (SC), France	Retrospective cohort	MSAP and SAP with SVT	Anticoagulant therapy (n=39)	Without anticoagulation (n=37)	No significant difference in cavernoma, mortality, LOS; ↑ bleeding
		Pagliari et al. (140)	2019	Tertiary (SC), Italy	Retrospective survey	AP with SVT	100 UI/kg b.i.d. at the diagnosis, with fondaparinux 7.5 mg/d, or vitamin K antagonist, or the NDOAC, upon discharge (n=16)	Without anticoagulation (n=11)	↑ Recanalization rate.
		Junare et al. (141)	2020	Tertiary (SC), India	Prospective study	AP with SVT	Heparin, subsequently, warfarin (n=12)	Without anticoagulation (n=12)	No significant difference in varices, collateral formation, recanalization and mortality.
		Saleh et al. (142)	2022	Tertiary (SC), UK	Database	AP with SVT	Warfarin, rivaroxaban, apixaban, or dabigatran (n=550)	Without anticoagulation (n=950)	No significant difference in varices, ascites, small intestine excision, splenic infarction, small bowel ischemia, liver failure; ↑ Risk of GI bleeding
		Thejasvin K et al. (143)	2022	Tertiary (SC), UK	Retrospective study	AP with SVT	Therapeutic Enoxaparin or Tinzaparin and Apixaban on discharge, or prophylactic thromboprophylaxis (n=74)	Without anticoagulation (n=35)	↑ Recanalization rate in patients with PV thrombus than in splenic vein thrombosis
		Oyón et al. (144)	2023	Tertiary (MC), Spain	*Post hoc* analysis of a prospective cohort study	AP with SVT	Anticoagulation group (n=33)	Without anticoagulation (n=27)	↑ SVT resolution rate
		Eltweri et al. (145)	2024	Tertiary (MC), UK	Retrospective study	AP with iSVT	Heparin, LMWH, warfarin, NOAC (n=32)	Without anticoagulation (n=56)	↑ Recanalization rate

AP, acute pancreatitis; APC, Activated Protein C system; SC, single center; SAP, severe acute pancreatitis; MODS, Multiple organ dysfunction syndrome; OF, organ failure; DIC, disseminated intravascular coagulation; rTM, recombinant thrombomodulin; WON, walled-off necrosis; JAAM score, Japanese Association for Acute Medicine score; MC, multicenter; ANC, acute necrotic collection; APFC, acute peripancreatic fluid collection; RCT, Randomized controlled trial; LOS, length of stay; CTSI, Computed Tomography Severity Index; MOF, Multiple organ failure; MSAP, moderately severe acute pancreatitis; PN, pancreatic necrosis; NIS, Nationwide Inpatient Sample; US, United States; AKI, acute kidney injury; ICU, Intensive Care Unit; UK, the United Kingdom; SVT, splanchnic vein thrombosis; LMWH, Low molecular weight heparin; NDOAC, novel oral anticoagulant; GI, gastrointestinal; PV, portal vein; iSVT, Isolated splenic vein thrombosis;

LMWH was administered by subcutaneous injection, while heparin was administered by intravenous. ↑, Decrease; ↓, Increase.

There are still many potential drug targets that are only being used in research, and seldom in clinical practice. In an animal study, ATIII (500 μg/kg) was injected intravenously 30 min before or after the induction of severe AP in rats, which in turn ameliorate SAP-induced kidney injury by inhibiting inflammation, oxidative stress, and apoptosis (149). Inhibitors of P-selectin has been proved to have the therapeutic potential on diseases linked to immunothrombosis and coagulopathy (85), NETs targeting might also reduce risk of thrombus formation in the context of infections (104, 105). Inhibition of P-selection or NET formation do attenuate OF and neutrophil recruitment in the inflamed pancreas (150) and improve survival by improving pancreatic microcirculation (82, 84, 151), thereby necessitating the translation of these findings into clinical trials of severe AP patients.

## Conclusion

**Figure 2:**
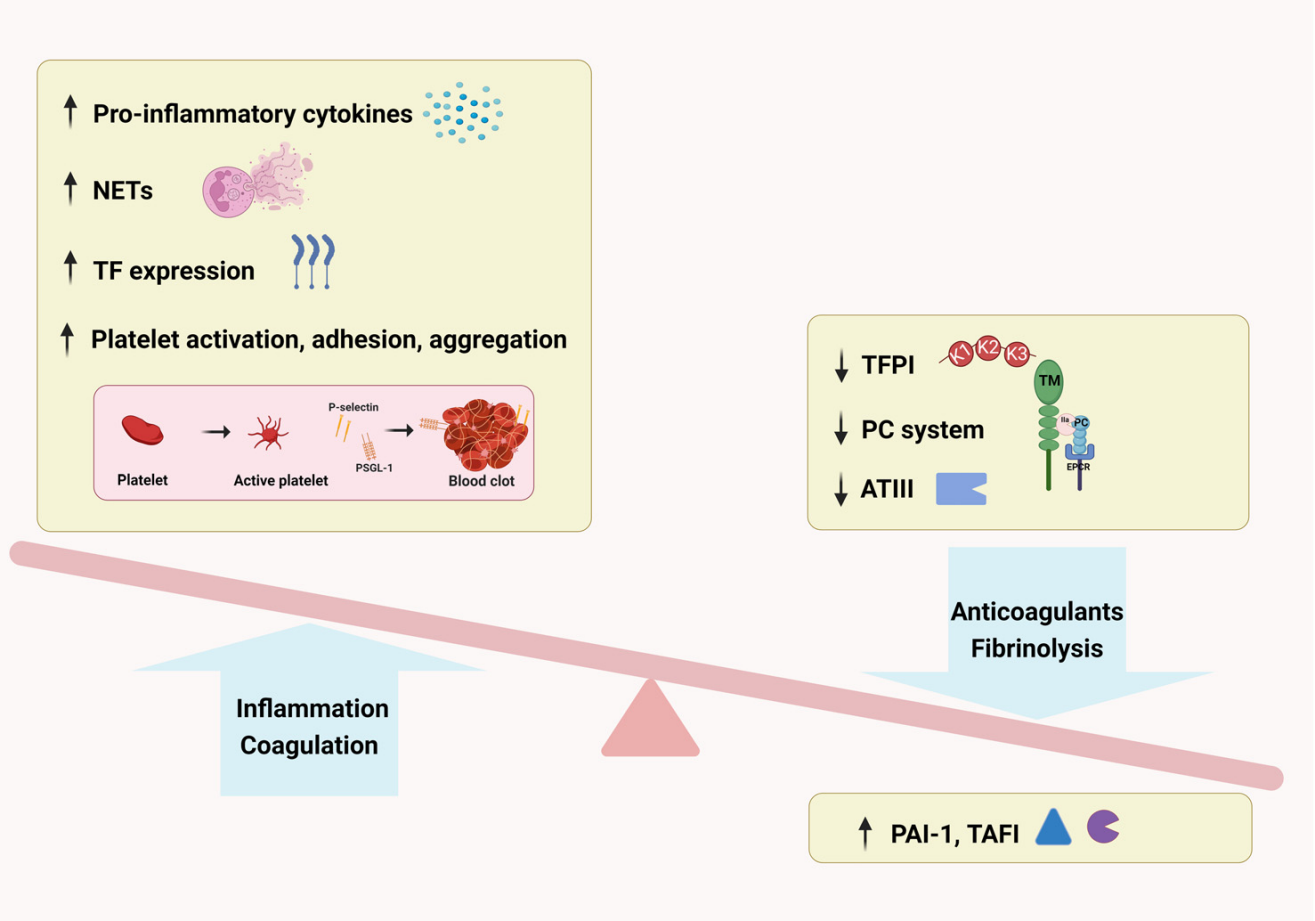
Imbalance of coagulation system in acute pancreatitis. Pro-inflammatory cytokines activate the coagulation cascade by neutrophil extracellular traps, tissue factor and platelets, downregulate crucial endogenous anticoagulant mechanisms through tissue factor pathway inhibitor, protein C system and antithrombin, concurrently dysregulate fibrinolytic mechanisms with plasminogen activator inhibitor type 1 and thrombin activated fibrinolytic inhibitor.

Taken together, pro-inflammatory cytokines activate the coagulation cascade, downregulate crucial endogenous anticoagulant mechanisms, and dysregulate fibrinolytic mechanisms (**Figure 2**). The coagulopathy has been ascribed a critical pathophysiological role in AP, arising from the complex interplay between innate immune, endothelial cells and platelets. Thus, a single-target therapy may be insufficient, requiring novel drugs. Large-scale clinical trials are needed to identify the appropriate drug and the adequate dose under various clinical situations.
